# Effects of repeated gravity changes during parabolic flight: Evidence of the need to assist space tourists to outer space

**DOI:** 10.1371/journal.pone.0320588

**Published:** 2025-04-23

**Authors:** Barbara Le Roy, Charles Martin-Krumm, Vincent Beauchamps, Adrien Jimenez, Louise Giaume, Sandrine Jacob, Aude Voilque, Ouamar Ferhani, Ellemarije Altena, Marion Trousselard

**Affiliations:** 1 Stress Neurophysiology Unit, French Armed Forces Biomedical Research Institute, Brétigny-sur-Orge, France; 2 CNES, Paris, France; 3 INSPIIRE UMR, Inserm, University of Lorraine, F-54000, Nancy, France; 4 École de Psychologues Praticiens, Catholic Institute of Paris, EA Religion, culture et société, Paris, France; 5 Fatigue and Vigilance Unit, French Armed Forces Biomedical Research Institute, Brétigny-sur-Orge, France; 6 AGM1, Brigade des Sapeurs-Pompiers de Paris, Paris, France; 7 Digital Innovation and Artificial Intelligence Department, French Armed Forces Biomedical Research Institute, Brétigny-sur-Orge, France; 8 University of Bordeaux, CNRS UMR, INCIA, Bordeaux France; 9 French Military Health Service Academy, Paris, France; Public Library of Science, UNITED STATES OF AMERICA

## Abstract

In the era of space tourism, walking in the steps of Neil Armstrong has never been more real. Future space tourists will have to face the harshness of the environment, especially the travel, and adapt quickly for their own safety. This issue raises both the question of preparation and the impact of such a journey on novice populations who have not been selected for their physical and cognitive abilities. The objectives of the study are (1) to investigate the impact of space travel on psychophysiological and sensory responses during a parabolic flight experience; (2) to assess recovery from this experience one week later; and (3) to evaluate the relevance of high parasympathetic functioning at baseline as a biomarker of adaptation. Seventeen healthy participants were enrolled in the 79th ESA Parabolic Flight Campaign on board the Airbus A310. Psychological, physiological, and sensory responses were measured at different times from the day before the 3h-flight (baseline) to one week after the flight (recovery). Labels were allocated to two groups according to their parasympathetic functioning at baseline: high parasympathetic (HP) profile and low parasympathetic (LP) profile. At the psychological level, those with an HP profile have a higher coping acceptation and a higher level of interoceptive awareness than the LP profile, except for sleep quality. At the physiological and exteroceptive level, they have a higher heart rate variability, preserved identification of odors and a predisposition to a more adaptive postural response postflight. Nevertheless, postural stability is affected in both profiles, particularly during visual deprivation, while their heart rate variability is increased in both linear and non-linear components. Nevertheless, our results reveal that the recovery constitutes a critical period. Flyers have a decrease of interoceptive awareness and emotions, especially the HP profile. Although the LP profile reported a better subjective sleep quality, both profiles decreased their sleep quality. These results raise the question of the risks that may be induced by space tourism. They highlight two major outcomes: (1) travel of future space tourists does not seem to be at risk as long as the individuals are qualified and fit for the flight and adaptation may be improved by targeting parasympathetic functioning; (2) level of experience has no impact on the psychophysiological and sensory responses. The results highlight the need to monitor the crews over several days and/or to include in the preparation a module allowing them to be prepared for the postflight period and the return to life on Earth. Beyond this, these results contribute to enriching our knowledge of the human challenge of confronting space travel constraints.

## 1. Introduction

Since the dawn of time, anyone who looks at the stars has aspired to touch them. With the advent of space tourism, many humans will soon be able to go beyond the boundaries of the Earth to experience being in space. Currently, the international space station (ISS) as well as the Chinese space station remain the only two manned stations in low-Earth orbit (i.e., altitude less than 2000 km from Earth) to explore the effects of such space journeys on human bodies. Spaceflight is associated with many physiological, sensory, and psychological effects that may lead to disturbances and impact the health and safety of crews [1–7]. More specifically, environmental factors (e.g., microgravity, accelerations, vibrations, cabin pressurization) may worsen previous pathologies or generate high levels of stress during suborbital spaceflight [8,9]. Harsh environments can impair the performance of astronauts due to stressors related to the environment and the mission [7,10–13]. In consequence, astronauts need to implement conservative regulations to maintain homeostatic status, especially concerning physiological factors, and in particular when faced with changes in gravity [4, 14–20]. Next to this, improved astronaut selection and mission preparation have reduced the occurrence of mental disorders among astronauts. While Shepanek [21] reported two psychiatric events on board the Mir space station, Garrett-Bakelman and colleagues [22] did not report such symptoms during the one-year mission of American astronaut Scott Kelly. Nevertheless, it is important to emphasize that the profile of space missions has changed, notably their length, since the ISS became a permanently manned station.

For many years, the literature emphasized the relevance of parabolic flights to better understand how the organism is able to adapt to this hostile environment, in particular to microgravity. The main interest of parabolic flights is two-fold: on the one hand, it can serve to verify the quality and success rate of space experiments before space missions and validate the results of space missions again afterwards. On the other hand, it provides an experimental laboratory to test the effects of gravity changes on cell cultures, and physiological factors in plants, animals and humans. Periods of weightlessness (0g) are combined with periods of normal gravity (1g), and hyper-gravity (1.8g). Parabolic flights induce a high psychological and physiological strain on flyers [23]. The impacts are bony [24], ocular [25] and cardiovascular [4,7,26] but several studies have focused on specific physiological [27,28], cerebral [12,29,30] and sensorial aspects [31,32]. More specifically, the project by Moser and Voica [33], which took place over several sessions, studied the adaptation of the vegetative nervous system before, during and after parabolic flights. Other projects have been carried out in the same years to evaluate the effect of microgravity on the cardiovascular system [34–36]. These projects are still relatively longstanding and showed that variations in heart size occur even during short periods of microgravity, and that some of the results concerning heart rate and aspects of the modulation of cardiovascular function by the autonomic nervous system (ANS) are similar to those observed during longer periods of spaceflight. Physiological individual characteristics are candidate factors for understanding stress adaptation mechanisms. One of these is based on the flexibility of the parasympathetic system at rest, measured by recording heart rate variability (HRV) [37–39]. This parasympathetic system is involved in stress regulation and participates in the individual’s adaptive response to external changes [40,41]. Therefore, HRV is an indicator of psychological and physiological stress, known to reflect both the parasympathetic and the sympathetic branches of the ANS. It is evaluated by measuring the variation in the interval between successive heartbeats (i.e., RR interval) [42,43]. Vagally-mediated HRV is commonly indexed by the root mean square of successive differences (RMSSD), and high-frequency (HF) [44,45].

Wollseiffen and collaborators studied cerebrovascular autoregulation as a determinant of neurocognitive performance [46,47]. Brummer et al. [48] confirm that parabolic flights induce a stress response that impacts physical states, cognition, and stress hormone release. Schneider et al. [49] have notably shown an association between stress hormone release, psychological and physical responses. An increase in cortisol, epinephrine, acetylcholine, and prolactin have been linked to a decrease in perceived physical state, psychological strain, as well as motivation. A decrease in norepinephrine has been linked to a decrease in motivation and psychological strain. More recently, Collado and collaborators [50] investigated the impact of parabolic flights on psychological states depending on the quality of adaptation. They differentiated maladaptative (i.e., at least one symptom) and adaptative (i.e., absence of symptoms) groups using the level of perceived motion sickness of participants during parabolic flights. Their results show that the maladaptive group have problems integrating sensory information and regulating mood, which led to difficulties in coping with the physical demands of the environment. Affective responses are likely to impact cognitive, physiological, and sensory functioning. These studies did not show an alteration of recovery and highlighted that individuals in fact recover immediately after landing [49].

Many studies have therefore been conducted in this flying laboratory, used to train astronauts for their space missions [51], but also relevant to explore the risks of space tourism journeys [52]. Recent launches from private compagnies (i.e., Blue Origin, Virgin Galactic, Space X, Axiom Space) have changed the rules of spaceflights, opening the way for space tourism. Competitiveness is at an all-time elevated level to secure a place in this market share, where the average entry ticket for launch is $1,000,000 [53]. The only transfers for an astronaut are currently approximately every six months from Earth to the ISS, and astronauts stay for a specific time in the ISS. Space tourism will involve journeys of at least a few hours and/or stays of varying lengths at space bases. Inter-base trips will also be considered. Considering that an astronaut requires several years of preparation to go into space, it seems urgent to understand the impact of these environments and challenges on an average individual. Institutions will have to prepare a candidate who does not meet the astronauts’ aptitude criteria. While astronauts will see their mission times increase, perform complex actions during their mission plans, and be exposed not only to the environmental factors of the space environment, but to isolation and confinement, private crews will be sent out for minimal flight durations, from a few hours at first to several days when commercial flights and the infrastructures to accommodate them will be sufficiently deployed. These crews will be able to enjoy the universe around them, within the limited constraints of the harsh environment. Private crews will not be selected for their physical, medical, and cognitive abilities but selected both for their desire to confront themselves to the limits of the Earth’s atmosphere and their financial capacities. Therefore, the selection as well as the training will not recover the same aim. An astronaut takes an average of 5–10 years to prepare for his or her first mission, while a space tourist’s preparation takes a few months at most. This new paradigm raises the question of risk assessment that must be carried out for short- and medium-term “tourist missions”. Private crews will be part of a unique experience that few human beings have ever had. Observing one’s “earthly” home beyond the boundaries of the atmosphere is no mean feat. Astronauts have long reported experiencing emotions so strong that they could lead to sensations beyond the real, mystical. Some of them have experienced profound psychic upheavals on their return to Earth. This needs to be assessed prior to these new types of missions in order to target individuals at risk.

Antuñano and collaborators [8] listed six domains/consequences that may occur during a tourist flight: inflight medical emergency, inflight death, alteration of health and security of passengers, compromise the protective equipment, emergency procedures, and jeopardize the safety of the flight. These risks start at the beginning of the space travel by considering the displacement from the Earth, i.e., the journey. This challenge raises the question of the impact of the flight on tourists’ adaptation during the space experience, and on their health after it. They cited different pathologies that may be of concern including psychiatric disorders, neurological disorders, sleep disturbances, addictions, cognitive impairments, vision, and hearing disabilities. These disorders may lead to an alteration of the behavior and performance of crews, resulting in an inability to operate efficiently for emergencies and to communicate effectively with the ground. Surprisingly, there is little emphasis on the impact of the journey itself. This impact on health for a non-astronaut population remains unknown, as do the repercussions several days postflight. This is a real challenge for the ambition to send private crews into space. Thus, we conducted a study on individuals not having received any selection and spatial training with the following objectives: (1) to investigate the impact of space travel on psychophysiological and sensory responses using a parabolic flight experience; (2) to assess recovery from a parabolic flight experience one week later; and (3) to evaluate the relevance of high parasympathetic functioning at baseline as a biomarker of adaptation.

## 2. Materials & methods

### 2.1. Design

The present study (ID-RCB: 2022-A01317-36) was approved by the Committee for the Protection of Individuals (CPP Ile de France XI, Montigny-le-Bretonneux, France) and was conducted according to the standards of the Declaration of Helsinki. After comprehensive verbal and written presentations, all participants gave their written consent to participate.

### 2.2. Participants

Health status of participants was confirmed by the delivery of a parabolic flight medical aptitude certificate as well as an assessment of clinical history and examination at inclusion.

Participants were healthy scientists (*n* = 7), physicians (*n* = 5), academic coordinators (*n* = 3), engineers (*n* = 2), and other professions (*n* = 1). The recruitment process took place between the 24^th^ and 26^th^ of October, 2022. One participant (from the scientific team) was excluded for medical reasons, leaving 17 subjects (four women and 13 men). The average age is 41.35 ± 8.95, ranging from 27 to 61 years. Among the four women, one (25.00%) was using contraception (i.e., a copper intrauterine device). None of the participants were taking medication or were smokers. Two participants (11.76%) have had a rip replacement or suffered from deafness in the right ear since the age of ten. Average height was 177.35 ± 6.83 meters, and weight was 69.71 ± 9.06 kilos. All participants had a spouse and nine participants (52.94%) had children. 12 (70.58%) reported having encountered personal (2.66 ± 1.37) or/and 10 (58.82%) professional (6.00 ± 5.04) major stressful events during their life.

Six (35.29%) already experienced extreme environments (e.g., survival at sea, expeditions in remote locations, mission in Antarctica, life simulation on Mars). 10 (58.82%) never experienced parabolic flight campaign, three (17.64%), and four (23.52%) participants had participated in at least 30 parabolic flights.

Table 1 Reports sociodemographic characteristics.

**Table 1:** Socio-demographic characteristics of participants.

Measurements	Microgravity data^*^
N	17
Mage	41.35 ± 8.95
Mheight	177.35 ± 6.83
Mweight	69.71 ± 9.06
Gender (women/men)	23.52%/76.47%
In couple/with children	100%/52.94%
Contraception	25.00%
Clinical medical history (i.e., hip replacement, deafness)	11.76%
Personal major stressful events	2.66 ± 1.37
Professional major stressful events	6.00 ± 5.04
Previous experience in extremes	35.29%
Parabolic flight experience (novice/beginner/expert)	58.82%/17.64%/23.52%

* Mean and standard deviation are reported when necessary. Other figures show the ratio of the number of subjects

### 2.3. Data collection

#### 2.3.1. Subjective measurements.

A 20-item sociodemographic questionnaire was developed to collect general information on the participant’s family situation, medical history, current health status, hobbies, familiarity with extreme environments and parabolic flights.

Psychology. The Coping Flexibility Scale (CFS) assesses coping flexibility defined as the ability to halt an ineffective coping strategy (i.e., evaluative coping) and to instead apply an alternative coping strategy (i.e., adaptive coping) (10 items) [54]. The Freiburg Mindfulness Inventory (FMI) evaluates mindfulness disposition (14 items) [55]. The scale is divided into two sub-factors that measure presence and acceptance without judgment. The Multidimensional Assessment of Interoceptive Awareness (MAIA) scale evaluates interoceptive awareness (32 items) [56]. The scale is divided into eight sub-factors that measure awareness of uncomfortable, comfortable, and neutral body sensations, the response to sensations of pain and discomfort, the ability to regulate attention to body sensations, and awareness of mind-body integration (i.e., noticing, not distracting, not worrying, attention regulation, emotional awareness, self-regulation, body listening, trusting). The Scale of Positive and Negative Experience (SPANE) assesses subjective feelings of positive and negative affect, based on how frequently they were experienced over the previous four weeks (12 items) [57]. The scale is divided into two sub-factors: positive and negative affect. The Sleep Quality Scale (SQS) measures sleep quality (1 item) [58]. The Perceived Stress Scale (PSS) assesses the subjective stress level (14 items) [59].

Extrasensors. The Postural Awareness Scale (PAS) assesses mind-body awareness, divided into two sub-actors that measure ease/familiarity with postural awareness and need for attention regulation with postural awareness (20 items) [60]. We added three clinical questions ourselves asking about levels of nausea, dizziness, vertigo during the flight (3 items).

#### 2.3.2. Physiological measurements.

Sleep activity was recorded the night before and the day after the flight using an actimeter (MotionWatch 8, CamNtech, Texas, USA) [61]. This device monitors sleep and wake duration, circadian rhythms, and physical activity. Common outputs such as Wakefulness After Sleep Onset (WASO) are reported here. A sleep diary was completed on awakening to collect subjective information on the quality of the night (i.e., feeling well-rested when waking up, having repetitive thoughts, experiencing physical indicators of stress, having had vivid dreams, and the frequency of waking up).

Heartbeat interval data (RR) were recorded for a ten-minute period, with subjects being in a sitting position. The cardiac biosignal was recorded using a Polar H10 pectoral chest belt (Polar, Finland), at a sampling frequency of 180 Hz. The literature confirms the reliability of the Polar H10 belt to measure HRV [62–66], and its use has been validated in a military population [67]. Furthermore, it has been shown to be as accurate as a reference ECG at rest, and during exercise [68]. This device’s low artifact rate makes it the best-available portable tool for recording cardiac activity, especially in studies in ecological environments. Three sensors pick up electrical signals from the heartbeat, which are sent in real time, via Bluetooth, to a software application [69].

The HRV analysis followed guidelines reported in [38,70], which take into account potential circadian variation, and used the *PyHRV* python library [71]. The following data were further assessed: weight; height; waist-to-hip ratio; smoking habits; most recent alcohol intake (>24h); most recent caffeinated (coffee/ tea) intake (>1h); most recent meal (>2h); most recent physical activity (>12h); and quality of sleep on the day of the experiment and the preceding day.

Raw ECG data were filtered between 3 and 45 Hz using a finite impulse response band-pass filter. The order of the filter was set at 54 (0.3 times the sampling frequency). R peaks were automatically detected using the *BioSPPy* python library [72]. A Hamilton segmentation was performed on the filtered signal, followed by R-peak correction with tolerance set to 0.05. R-waves were manually examined to ensure correct detection. If an ECG sequence was overly noisy when visualizing the superposition of all QRS complex, the time interval was manually removed to improve data quality. RR intervals were automatically detected with the *hrvanalysis* module using linear interpolation, and manually corrected for artifacts and ectopic beats.

Time domain analysis. Time domain HRV metrics included mean RR (the mean inter-beat interval), SDNN (Standard Deviation of the Normal-to-Normal RR interval), RMSSD, and pNN50 (percentage of adjacent NN intervals that differ from each other by more than 50 ms).

Frequency domain analysis. Frequency domain HRV metrics complemented time domain metrics and included oscillatory components of heart rate dynamics. Spectral density was estimated using Welch’s method: low frequencies (LF, sympathetic and parasympathetic activity) in the range 0.04–0.15 Hz, and high frequencies (parasympathetic activity) in the range 0.15–0.4 Hz.

Nonlinear analysis. Nonlinear HRV metrics reflect dynamic and chaotic internal states that other metrics cannot reflect. The most representative metrics were used: the Poincaré plot (i.e., graphical representation of the correlation between successive inter-beat intervals), SD1 (i.e., standard deviation of instantaneous inter-beat interval variability), SD2 (i.e., standard deviation of continuous, long-term RR variability), α1 (i.e., detrended fluctuation analysis self-similarity parameter that represented short-term fluctuations), α2 (i.e., detrended fluctuation analysis self-similarity parameter that represented long-term fluctuations), SampEn (i.e., sample entropy achieving the regularity and complexity of time series).

#### 2.3.3. Exteroceptive measurements.

Olfaction. The european test of olfactory capabilities (ETOC) assesses olfactory sensitivity. Individuals are asked to select a bottle (there are 16 sets of four bottles) that contains a specific odor (a discrimination task) and state the nature of this odor (an identification task) [73]. In our experiment, participants were also asked to evaluate the hedonic value of the detected odor to complement the ETOC.

Proprioception. Posturography was assessed using a stabilometric static platform (Stabilotest). The acquisition frequency is 40 Hz. Subjects were asked to stand for one minute with their eyes open, and for one minute with their eyes closed. Among the numerous metrics used to characterize postural stability reported in the literature [74,75], we assessed the most frequent measures: elliptic surface (mm^2^); LFS (i.e., length ratio of the surface, mm^-1^); asymmetries using logarithm (mm) along sagittal (antero-posterior) and frontal (medio-lateral) planes; slope (degree); average speed (mm/s); variance speed (mm/s); NA02 (i.e., maximum distance covered by the center of pressure).

### 2.4. Experimental design

The microgravity experiment was run during the last week of October 2022 at Novespace (Bordeaux-Merignac International Airport, France).

**Fig 1:**
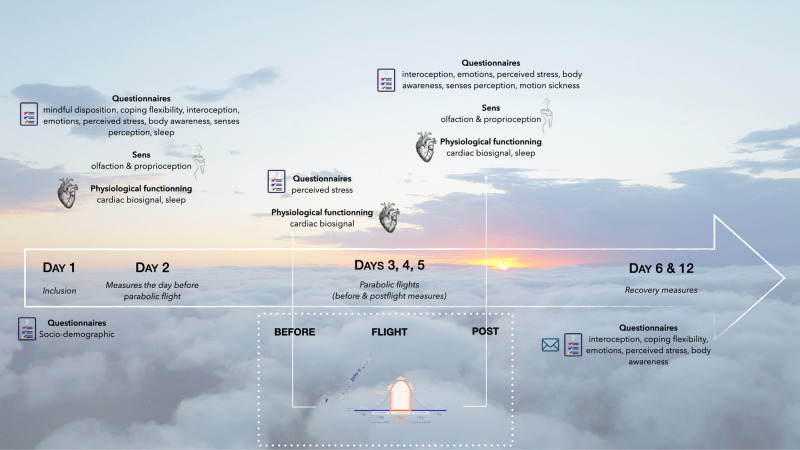
Overview of measures collected during parabolic flight campaign.

**Fig 2:**
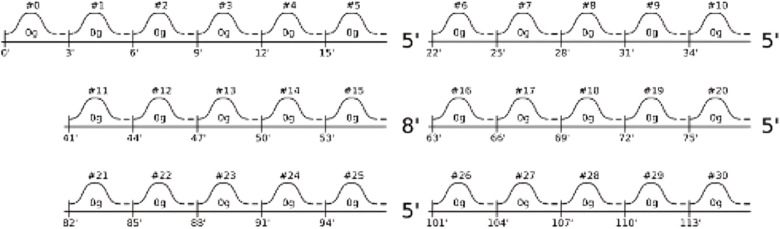
Parabolas’ composition during a parabolic flight in microgravity.

**Fig 3:**
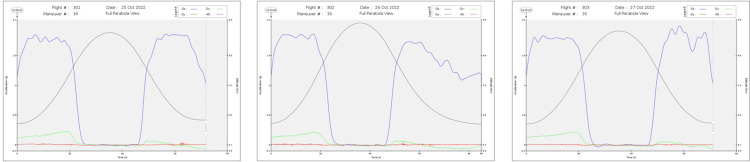
Accelerometer data of parabola 16 of the 79^th^parabolic flight campaign.

On Monday, baseline measures were performed (i.e., psychology, physiology, sensory measurements, except for motion sickness) after verification of the inclusion criteria. The parabolic flights were organized on Tuesday, Wednesday, and Thursday (Fig 1). Each participant participated to one flight only. Parabolic flights took place on board the Airbus A310 ZERO-G, a plane specially modified for microgravity and hypergravity situations. The flight runs using 30 parabolas (i.e., three to four hours). One parabola maneuver is composed of a pull-up, a microgravity phase, and a pull-out (Fig 2). The duration of the microgravity phase last about 21 seconds. Data from accelerometers for each flight is shown in Fig 3.

**Fig 4:**
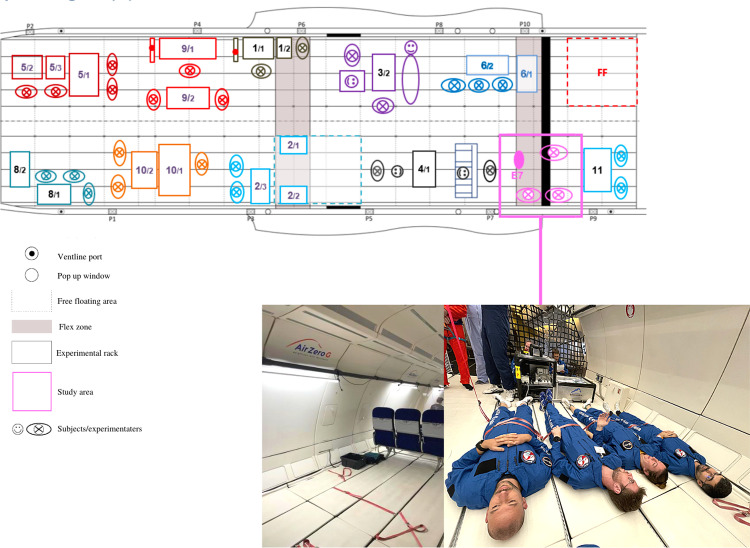
Experimental setting on board the Airbus A310 in microgravity.

Participants were seated in a relaxed position and were firmly and comfortably strapped to the floor in order to avoid artifacts caused by stabilization movements (Fig 4). As soon as the flight landed, postflight measurements were performed (i.e., psychology, physiology, sensory measurements). The day after the flight, as well as seven days later, the subjects received an e-mail link to a questionnaire to assess the recovery of the psychological state (i.e., sleep, emotions, coping flexibility, interoception, postural awareness, subjective perception of stress and senses).

Baseline assessments were performed the day before the flight (D−1). Preflight (pre-f) and postflight (pos-f) assessments were conducted at 7 am before boarding and just after landing. Recovery assessments took place the day after the parabolic flight (D+1), and one week after (D+7). Psychological and exteroceptive data were assessed at D−1 and post-f. HRV was recorded at D−1, pre-f, and post-f for the microgravity campaign. Sleep activities were recorded twice: the night before and the day after the parabolic flight (D−1 and D+1). Motion sickness was evaluated at pre-f and post-f.

### 2.5. Statistical methods – analysis

Statistics were computed for all outcome measures. Data analyses were performed with JASP (Amsterdam, version 0.16.3), an open-source software package that is used for both classical and Bayesian analyses. Descriptive statistics were expressed as mean ± SD. The Shapiro–Wilk test was used to determine whether data were normally distributed. When the analysis was significant, effect sizes were reported. Stress adaptability was evaluated using one baseline HRV metric (i.e., RMSSD), linked to the parasympathetic branch of the ANS. Thus, the median was used to distinguish our population. In this sense, we consider the hypothesis that we have different groups based on their parasympathetic activity at rest. This statement conducts to two different profiles: the HP profile have a high parasympathetic functioning and the LP profile have a low parasympathetic functioning at baseline. To evaluate the difference between HP and LP profiles at baseline, ANOVA one-way analysis or Kruskal-Wallis test (i.e., depending on the normality of the distribution) were performed. Psychological, physiological, and exteroceptive states were evaluated using ANOVA repeated-measures analyses. Holm *post hoc* analyses were performed when the *p*-value was significant. Bayesian analyses were performed, by applying equivalent analyses for ANOVA repeated-measures analyses. The Bayesian Factor was calculated if no significant effect was detected. A low value provides support for the null hypothesis, and a high value indicates evidence in favor of the alternative hypothesis (Supplementary Material in S1 Text).

For all analyses, statistical significance was set at *p* <.05. A *p*-value between.05 and.07 was considered as evidence of a trend.

## 3. Results

D−1 measurements for all participants per groups are presented in Tables 2–4 to 4 (Supplementary Material in S1 Text). No difference was found between the two profiles based on the RMSSD at D−1for health and psychological measurements, apart from coping acceptance, subjective stress, the interoceptive sub-factor “not distracting”, and a trend for the interoceptive sub-factor “trusting” (Table 2) and for. Further, no differences were found for proprioceptive measurements, apart from the slope in the opened eyes condition and the NA02 front-to-rear in the closed eyes condition (Table 3). Concerning physiological measurements at D−1, the groups based on the RMSSD level were different on SDNN, pNN50, LF, HF, SD1, SD2, SD ratio and α2 (Table 4). Previous parabolic flight experience did not highlight differences on any measures recorded. Moreover, motion sickness of participants was assessed pre-f and post-f but no significance was found between participants, whatever the parasympathetic functioning profile.

**Table 2:** Baseline health & psychological measurements among parasympathetic profiles.

	LP profile	HP profile	*p*-value^*^
**CFS**			
E	4.000 ±.000	5.400 ± 1.517	.922
A	4.000 ± 1.414	5.600 ± 2.408	.433
**FMI**			
PR	18.875 ± 1.885	19.333 ± 1.581	.594
AC	19.375 ± 3.777	22.444 ± 2.242	.056
**MAIA**			
N	2.875 ± 1.157	3.694 ±.497	.161
ND	1.563 ±.826	2.907 ±.972	.008
NW	2.700 ±.701	3.156 ±.488	.137
AR	3.107 ±.735	3.286 ± 1.020	.688
EA	3.225 ± 1.000	3.244 ±.871	.966
SR	2.688 ±.989	3.222 ±.701	.214
BL	1.792 ±.975	2.667 ± 1.130	.110
T	3.333 ± 1.321	4.407 ±.703	.077
**SPANE**			
N	3.896 ±.281	4.222 ±.464	.205
P	2.354 ±.594	2.167 ±.577	.520
**SQS**			
SQ	6.750 ± 1.982	5.111 ± 2.315	.140
**PSS**			
S	63.853 ± 6.582	70.109 ± 5.608	.051
**PAS**			
PA	14.039 ± 2.947	16.126 ± 2.086	.109
NA	45.083 ± 4.846	49.407 ± 3.741	.056

*Note*. LP = low parasympathetic functioning; HP = high parasympathetic functioning; E = evaluation; A = adaptation; PR = presence; AC = acceptation; N = noticing; ND = not distracting; NW = not worrying; AR = attention regulation; EA = emotional awareness; SR = self-regulation; BL = body listening; T = trusting; N = negative emotions; P = positive emotions; SQ = sleep quality; S = subjective stress; PA = postural awareness; NA = need to regulate attention.

* *p*-value used in the analysis of effects (ANOVA or Kruskall-Wallis test). Means and standard deviation are show for each variable.

**Table 3:** Baseline exteroceptive measurements among parasympathetic profiles.

	LP profile	HP profile	*p*-value^*^
**ETOC**			
D	15.286 ±.756	15.222 ± 1.641	.096
I	12.429 ± 1.988	13.000 ± 2.212	.592
H	82.036 ± 5.365	78.764 ± 9.676	.437
**Proprioception**			
OElg	626.019 ± 44.702	661.381 ± 167.313	.572
OEe	169.649 ± 53.121	167.766 ± 78.535	.955
OEs	1.469 ±.224	.302 ± 1.486	.045
OElfs	4.062 ± 1.438	5.124 ± 3.134	.394
OEas	11.481 ± 1.369	11.885 ± 2.928	.727
OEsv	45.633 ± 14.480	52.546 ± 28.356	.545
OElgLtR	345.825 ± 28.591	330.256 ± 71.221	.573
OErLtR	16.425 ± 2.023	16.922 ± 5.778	.821
OEvLtR	8.363 ± 2.277	8.731 ± 4.825	.847
OEsvLtR	45.633 ± 14.480	52.546 ± 28.356	.545
OEna02LtR	24.557 ± 15.922	21.379 ± 7.851	.603
OElgFtR	436.238 ± 51.864	492.111 ± 148.116	.328
OErFtR	21.512 ± 4.489	21.733 ± 6.308	.936
OEvFtR	19.926 ± 11.523	20.515 ± 13.126	.923
OEsvFtR	109.119 ± 37.087	137.814 ± 83.880	.387
OEna02FtR	20.438 ± 8.269	17.807 ± 5.343	.443
CElg	902.800 ± 258.108	824.258 ± 239.449	.525
CEe	282.588 ± 180.709	197.308 ± 100.756	.241
CEs	-.303 ± 1.535	-.399 ± 1.470	.897
CElfs	3.781 ± 1.180	4.968 ± 2.422	.228
CEas	16.272 ± 4.944	15.292 ± 4.353	.670
CEsv	125.954 ± 98.196	92.639 ± 59.180	.404
CElgLtR	466.137 ± 107.097	379.233 ± 101.603	.107
CErLtR	21.650 ± 5.842	17.544 ± 5.259	.148
CEvLtR	15.768 ± 7.693	10.305 ± 7.945	.172
CEsvLtR	137.210 ± 72.582	89.832 ± 45.181	.122
CEna02LtR	25.182 ± 3.273	24.883 ± 7.208	.916
CElgFtR	664.575 ± 225.367	644.833 ± 204.774	.852
CErFtR	26.975 ± 11.590	26.478 ± 7.362	.916
CEvFtR	26.378 ± 20.211	24.214 ± 15.553	.807
CEsvFtR	274.986 ± 223.157	253.594 ± 179.636	.830
CEna02FtR	28.621 ± 9.117	20.729 ± 6.470	.055

*Note*. LP = low parasympathetic functioning; HP = high parasympathetic functioning; D = detection; I = identification; H = hedonic value; OE= opened eyes; lg = logarithm; e = elliptic surface; s = slope; lfs = length ratio of the surface; as = average speed; sv = speed variance; r = range; v = variance; na02 = amplitude of postural oscillations in X and Y; LtR = left to right; FtR = front to rear; CE = closed eyes

* *p*-value used in the analysis of effects (ANOVA or Kruskall-Wallis test). Means and standard deviation are show for each variable.

**Table 4:** Baseline physiological measurements among parasympathetic profiles.

	LP profile	HP profile	*p*-value^*^
**Temporal domain**			
Mean RR	776.582 ± 111.991	824.399 ± 122.463	.416
SDNN	40.288 ± 10.866	70.051 ± 15.654	<.001
RMSSD	17.472 ± 4.084	43.581 ± 11.138	<.001
pNN50	1.664 ± 2.132	23.191 ± 10.317	<.001
**Frequency domain**			
LF	581.156 ± 511.736	3198.754 ± 2357.656	.001
HF	120.895 ± 52.020	591.278 ± 288.658	<.001
LF/HF ratio	4.434 ± 2.372	6.785 ± 6.814	.370
**Non linear domain**			
SD1	12.354 ± 2.888	30.816 ± 7.876	<.001
SD2	55.489 ± 15.151	93.857 ± 21.446	<.001
SD ratio	4.465 ±.642	3.139 ±.614	<.001
a1	1.401 ±.129	1.272 ±.192	.130
a2	1.011 ±.144	.651 ±.232	.003
SampEn	1.093 ±.402	1.198 ±.324	.559

*Note*. LP = low parasympathetic functioning; HP = high parasympathetic functioning

* *p*-value used in the analysis of effects (ANOVA or Kruskall-Wallis test). Means and standard deviation are show for each variable.

### 3.1. Psychological evolution shaped by parabolic flights

*Coping*. There is a significant effect of time on evaluation [F (2,10) = 4.888, *p* =.033, *η*^*2*^ =.173]. *Post hoc* analyses show that the level of coping evaluation was higher at D+1(*p* =.056), and at D+7 (*p* =.056) compared to D−1.

*Mindfulness disposition*. There is a tendency to a significant group effect on acceptance [F (1,15) = 3.882, *p* =.068, *η*^*2*^ =.103]. Participants with a HP profile would have a higher acceptance than those with a low LP profile.

*Interoception*. There are significant time effects for “not distracting” [F (3,45) = 2.946, *p* =.043, *η*^*2*^ =.028] and group effect for “not distracting” [F (1,15) = 7.266, *p* =.017, *η*^*2*^ =.265], “body listening” [F (1,15) = 4.594, *p* =.049, *η*^*2*^ =.217] and “trusting” [F (1,15) = 7.266, *p* =.017, *η*^*2*^ =.265]. A trend to a time effect was identified for attention regulation [F (3,45) = 2.627, *p* =.062, *η*^*2*^ =.021]. *Post hoc* analyses reveal that participants ignored or distracted themselves from sensations of pain or discomfort (*p* =.044) and tended to decrease their ability to sustain and control attention to their body sensations (*p* =.066) at D+1 compared to D−1. Also, those with a HP profile did not ignore or distract themselves from sensations of pain or discomfort, experienced their body as a safe and trustworthy place, tended to actively listen to their body for insight compared to those with a LP profile. For emotional awareness, the Bayesian analysis supports the alternative hypothesis for the group (*p* =.618, BF10 = 1.822). In conclusion, flyers who have a HP profile tend to show a better awareness of the connection between body sensations and emotional states compared to those with a LP profile.

*Affects*. There are significant time effects [F (3,45) = 76.899, p <.001, η2 =.722] and time*group effect [F (3,45) = 5.475, p =.003, η2 =.051] on positive affect. There is also a significant time effect [F (3,45) = 123.705, *p* <.001, *η*^*2*^ =.769] on negative affect, in the absence of an interaction effect. *Post hoc* analyses revealed that participants have lower positive and higher negative affect at D+1 and D+7 compared to D−1 and post-f (*p* <.001). Also, those with an LP profile have lower positive affect at D+1 and D+7 compared to D−1 and post-f (*p* <.001). Those with an HP profile have lower positive affect at D+1 and D+7 compared to D−1 and post-f (*p* <.001). Participants with an HP profile showed a decrease of their positive affect at D+1 and D+7 compared to those with a LP profile at D−1 and post-f (*p* <.001). Participants with a LP profile showed a decrease of their positive affect at D+1 and D+7 compared to those with a HP profile at D−1 and post-f (*p* <.001).

*Subjective sleep.* Subjective measures revealed a significant time effect on sleep quality [F (3,45) = 3.394, p =.026, η2 =.118] and repetitive thoughts before bed, [F (1,11) = 10.750, p =.007, η2 =.088], and a time*group effect for physical indicators of stress [F (1,11) = 6.605, p =.026, η2 =.180] as well as a group effect on sleep quality [F (1,15) = 10.335, p =.006, η2 =.145], and physical indicators of stress [F (1,11) = 4.721, p =.053, η2 =.125]. Trends for a time effect were found for feeling well-rested when waking up [F (1,11) = 3.743, p =.079, η2 =.118] and physical indicators of stress [F (1,11) = 3.736, p =.079, *η*^*2*^ =.102]. *Post hoc* analyses showed that those with an LP profile have higher physical indicators of stress before bed compared to those with an HP profile at pre-f (*p* =.017), at pre-f compared to D+1 (*p* =.043), and at pre-f compared to those with a HP profile at D+1 (*p* =.041). Moreover, participants showed higher sleep quality (*p* <.034) at D+7 compared to D−1. Overall, flyers tend to have less repetitive thoughts and physical indicators of stress before going to bed, and higher levels of feeling well-rested when waking up in the morning, at D+1 compared to pre-f. Also, those with an HP profile have fewer physical indicators of stress before going to bed but lower sleep quality compared to those with an LP profile.

*Subjective stress*. There was a significant effect of time on stress [F (2,54) = 3.716, *p* =.031, *η*^*2*^=.121]. *Post hoc* analyses revealed that participants tended to perceive less stress at post-f (*p* =.063).

*Postural awareness*. There was a significant effect of time towards “need for attention regulation” with postural awareness [F (3,45) = 3.180, *p* =.033, *η*^*2*^ =.016]. *Post hoc* analyses showed that participants tended to increase their need for attention regulation with postural awareness (*p* =.098) at D+1 compared to D−1.

### 3.2. Physiological evolution shaped by parabolic flights

*Sleep monitoring*. The monitoring night shows a significant time effect for WASO [F (1,9) = 7.391, p =.024, η2 =.120], sleep efficiency [F (1,9) = 6.744, p =.029, η2 =.112], actual sleep time [F (1,9) = 10.944, p =.009, η2 =.153] and actual wake time [F (1,9) = 7.942, p =.020, η2 =.094]. Flyers have more nighttime awakenings, a lower sleep efficiency, and sleep duration at D+1 compared to pre-f.

*HRV*. The following significant differences are found: there are significant time effect for RR intervals [F (2,30) = 14.251, p <.001, η2 =.123], SDNN [F (2,30) = 5.352, p =.010, η2 =.075], RMSSD [F (2,30) = 13.602, p <.001, η2 =.140], pNN50 [F (2,30) = 17.156, p <.001, η2 =.142], HF [F (2,30) = 5.673, p =.008, η2 =.130], SD1 [F (2,30) = 13.603, p <.001, η2 =.140], SD2 [F (2,30) = 3.941, p =.030, η2 =.051], SD ratio [F (2,30) = 4.741, p =.016, η2 =.115], α1 [F (2,30) = 5.984, p =.007, η2 =.124]; time*group effect for α2 [F (2,30) = 5.975, p =.007, η2 =.064], group effect for SDNN [F (1,15) = 11.541, p =.004, η2 =.310], RMSSD [F (1,15) = 14.170, p =.002, η2 =.338], pNN50 [F (1,15) = 16.039, p =.001, η2 =.372], LF [F (1,15) = 6.098, p =.026, η2 =.238], HF [F (1,15) = 8.962, p =.009, η2 =.172], SD1 [F (1,15) = 14.170, p =.002, η2 =.338], SD2 [F (1,15) = 12.087, p =.003, η2 =.337], SD ratio [F (1,15) = 11.915, p =.004, η2 =.221], α2 [F (1,15) = 6.482, p =.022, η2 =.233]. Trends are identified for a time effect for SampEn [F (2,30) = 3.063, p =.062, η2 =.077] and for a time*group effect for HF [F (2,30) = 2.855, *p* =.073, *η*^*2*^ =.066].

*Post hoc* analyses revealed that participants have higher RR intervals, RMSSD, pNN50, SD1, SD2 at post-f compared to D−1 and pre-f (*p* <.001), a higher SDNN at post-f compared to D−1 (*p* =.029) and pre-f (*p* =.015), a higher HF at post-f compared to D−1 (*p* =.016) and pre-f (*p* =.017), a higher SD ratio at post-f compared to D−1 and pre-f (*p* =.033), a higher α1 at post-f compared to D−1 (*p* =.012) and pre-f (*p* =.016). Those with a HP profile increase their HF at post-f compared to D−1 (*p* =.010), pre-f (*p* =.014) and compared to those with a LP profile activation at D−1 (*p* =.001), pre-f (*p* =.002). They have a lower α2 at D−1compared to those with a LP profile at pre-f (*p* =.047). At post-f, those with a HP profile increase their HF compared to those with a LP profile (*p* =.006). At D−1, flyers with a HP profile have a lower α2 compared to those with a LP profile (*p* =.015). Also, the HP profile have a higher SDNN, RMSSD, pNN50, LF, SD1, SD2 and a lower α2 compared to those with a LP profile. Participants tend to increase their SampEn at post-f compared to D−1 (*p* =.063).

### 3.3. Exteroceptive evolution shaped by parabolic flights

*Olfaction.* A significant time effect is found for detection [F (1,14) = 6.168, *p* =.026, *η*^*2*^ =.044]. Participants increase their ability to identify odors (*p* =.098) at post-f compared to D−1.

*Proprioception*. Significant differences were found in both open and closed eyes conditions. Specifically, in the open eyes condition, there is a significant time*group effect for slope [F (1,15) = 6.051, p =.027, η2 =.155]. Trends for a group effect were identified for the logarithm left to right [F (1,15) = 3.726, p =.073, η2 =.105] and for the variance speed left to right [F (1,15) = 3.800, p =.070, η2 =.105]. Post hoc analyses show that the LP profile tends to have a higher slope than the HP profile in opened eyes condition at D−1 (*p* =.078) whereas the HP profile tends to increase their slope at post-f compared to D−1 (*p* =.078). Moreover, the HP profile tend to have a lower logarithm left to right and a higher speed variance compared to the LP profile.

In the closed eyes condition, there are significant time effects with an increase of the slope [F (1,15) = 15.279, p =.001, η2 =.244], a decrease of the LFS (i.e., energetic expenditure or length ratio of the surface) [F (1,15) = 5.479, p =.033, η2 =.061] post-f compared to D−1, and a significant group effect for the logarithm left to right [F (1,15) =.810, *p* =.030, *η*^*2*^ =.192]. The HP profile has a lower logarithm left to right compared to the LP profile.

Table 5 presents a summary of these psychological, physiological, and sensory differences following the experiment.

**Table 5:** Summary of the impact of parabolic flight on body functioning among parasympathetic profiles.

	Baseline		Pre-f		Post-f		D+1		D+7		*p*-value*
	LP profile	HP profile	LP profile	HP profile	LP profile	HP profile	LP profile	HP profile	LP profile	HP profile	
**CFS**											
E^*a*^	4.000 ±.000	5.400 ± 1.517					7.500 ±.707	6.000 ± 2.449	7.000 ± 1.414	6.200 ± 2.683	.033
*FMI*											
A^*c*^	19.375 ± 3.777	22.444 ± 2.242					20.000 ± 3.071	21.889 ± 2.028	19.625 ± 3.021	22.000 ± 2.963	.068
**MAIA**											
ND^*a,c*^	1.563 ±.826	2.907 ±.972			1.792 ±.589	2.519 ±.891	1.458 ±.452	2.204 ±.964	1.604 ±.684	2.519 ±.797	.017
AR^*a*^	3.107 ±.735	3.286 ± 1.020			2.586 ±.924	3.270 ±.679	2.500 ± 1.198	3.175 ±.874	2.714 ± 1.091	2.714 ± 3.381	.062
BL^*c*^	1.792 ±.975	2.667 ± 1.130			1.708 ± 1.046	2.741 ± 1.038	1.958 ± 1.046	3.037 ± 1.230	2.000 ± 1.182	3.000 ± 1.236	.049
T^*c*^	3.333 ± 1.321	4.407 ±.703			3.125 ± 1.447	4.370 ±.696	3.417 ± 1.456	4.407 ±.596	3.458 ± 1.553	4.556 ±.726	.017
**SPANE**											
N^*a*^	3.896 ±.281	4.222 ±.464			4.062 ±.333	4.389 ±.449	2.625 ±.469	1.963 ±.790	2.625 ±.469	1.963 ±.790	.003
P^*a,c*^	2.354 ±.594	2.167 ± 577			2.417 ±.398	2.056 ±.507	4.063 ±.377	3.833 ±.449	4.063 ±.377	3.833 ±.449	<.001
**SQS**											
SQ^*a,c*^	6.750 ± 1.982	5.111 ± 2.315			7.250 ±.707	5.556 ± 1.424	7.625 ± 1.598	6.667 ± 1.658	8.250 ± 1.282	6.889 ± 1.900	.006
**PSS**											
S^*a*^	30.000 ± 3.117	28.778 ± 3.032			30.625 ± 2.973	28.556 ± 3.087	30.875 ± 3.482	28.000 ± 3.464	30.875 ± 5.194	28.333 ± 3.808	.031
**PAS**											
PA^*a*^	22.750 ± 5.898	25.444 ± 7.091			22.500 ± 7.764	26.444 ± 6.327	24.250 ± 7.166	27.778 ± 6.515	23.500 ± 7.426	28.222 ± 6.220	.033
**PS**											
SQ^*a,c*^	6.750 ± 1.982	5.111 ± 2.315	7.250 ±.707	5.556 ± 1.424			7.625 ± 1.598	6.667 ± 1.658	8.250 ± 1.282	6.889 ± 1.900	.026
RT^*a*^			2.597 ± 2.538	1.221 ± 2.155			.986 ± 1.868	.498 ± 1.058			.007
PIS^*a,b,c*^			2.735 ± 2.663	.130 ±.214			.245 ±.241	.482 ± 1.064			.026
WRw^*a*^			5.133 ± 1.156	5.041 ± 1.728			5.554 ± 2.089	6.726 ±.685			.079
**ETOC**											
D^*a*^	12.429 ± 1.988	13.000 ± 2.121			13.714 ± 1.976	13.333 ± 1.871					.026
**Actimeter**											
WASO^*a*^			31.167 ± 16.241	46.400 ± 10.621			46.667 ± 29.412	61.000 ± 21.131			.024
SE^*a*^			89.361 ± 4.828	85.021 ± 7.104			85.210 ± 6.708	79.906 ± 8.080			.029
AST^*a*^			91.996 ± 4.389	88.192 ± 3.482			86.437 ± 6.798	85.670 ± 4.357			.009
AWT^*a*^			8.004 ± 4.389	11.808 ± 3.483			12.082 ± 7.738	14.330 ± 4.357			.020
**Proprioception**											
OEs^*b*^	1.469 ±.224	.302 ± 1.486			1.129 ± 1.074	1.548 ±.019					.027
OElLtR^*c*^	345.825 ± 28.591	330.256 ± 71.221			374.425 ± 95.218	300.233 ± 59.135					.073
OEsvLtR^*c*^	70.013 ± 13.607	63.629 ± 32.025			89.666 ± 47.646	54.894 ± 20.500					.070
CEs^*a*^	-.303 ± 1.535	-.399 ± 1.470			.910 ± 1.155	1.199 ± 1.011					.001
CElfs^*a*^	3.781 ± 1.180	4.968 ± 2.422			3.480 ± 1.822	3.510 ± 1.390					.033
CElLtR^*c*^	466.137 ± 107.097	379.233 ± 101.603			455.337 ± 120.130	358.233 ± 66.972					.030
**HRV**											
Mean RR^*a*^	776.582 ± 111.991	824.399 ± 122.463	775.481 ± 103.618	805.321 ± 143.557	851.679 ± 121.769	945.870 ± 181.081					<.001
SDNN^*a,c*^	40.288 ± 10.866	70.051 ± 15.654	35.987 ± 14.426	69.040 ± 41.633	52.720 ± 13.221	89.112 ± 31.862					.004
RMSSD^*,a,c*^	17.472 ± 4.084	43.581 ± 11.138	19.354 ± 3.420	42.652 ± 24.936	32.489 ± 13.123	67.313 ± 31.217					.002
pNN50^*,a,c*^	1.664 ± 2.132	23.191 ± 10.317	1.716 ± 1.318	21.209 ± 19.341	12.603 ± 10.853	42.034 ± 22.861					.001
LF^*,a,c*^	581.156 ± 511.736	3198.754 ± 2357.656	621.271 ± 505.540	3283.969 ± 3573.229	1134.826 ± 914.672	4188.095 ± 4354.523					.026
HF^*,a,b,c*^	120.895 ± 52.020	591.278 ± 288.658	162.992 ± 81.660	655.102 ± 571.075	369.769 ± 349.655	1969.084 ± 1931.997					.009
SD1^*a,c*^	12.354 ± 2.888	30.816 ± 7.876	13.685 ± 2.418	30.159 ± 17.631	22.973 ± 9.279	47.597 ± 22.073					.002
SD2^*a,c*^	55.489 ± 15.151	93.857 ± 21.446	56.192 ± 11.782	99.083 ± 49.255	70.567 ± 17.609	116.082 ± 40.736					.003
SD ratio^*a,c*^	4.465 ±.642	3.139 ±.614	4.174 ±.859	3.474 ±.590	3.479 ± 1.303	2.733 ±.851					.004
α1^*a*^	1.401 ±.129	1.272 ±.192	1.349 ±.209	1.287 ±.202	1.224 ±.297	1.053 ±.351					.007
α2^*b,c*^	1.011 ±.144	.651 ±.232	.963 ±.180	.767 ±.221	.881 ±.112	.794 ±.242					.022
SampEn^*,a,c*^	1.093 ±.402	1.198 ±.324	1.336 ±.444	1.121 ±.249	1.317 ±.323	1.447 ±.343					.062

*Note*. LP = low parasympathetic functioning; HP = high parasympathetic functioning; E = evaluation; A = acceptation; ND = not distracting; AR = attention regulation; BL = body listening; T = trusting; N = negative emotions; P = positive emotions; PS = perceived sleep; S = subjective stress; PA = postural awareness; SQ = sleep quality; WASO = wake actual sleep onset; SE = sleep efficiency; AST = actual sleep time; AWT = actual wake time; D = detection; OEs = slope in opened eyes condition; OElLtR = logarithm left to right in opened eyes condition; OEsvLtR = speed variance left to right in opened eyes condition; CEs = slope in closed eyes condition; CElfs = length ratio of the surface in closed eyes condition; CElLtR = logarithm left to right in closed eyes condition.

*p-value used in the analysis of effects. Means and standard deviations are shown for each variable. All variables were recorded at four times, appart from proprioception where baseline and recovery were collected. Only significant interactions were reported (*p* <.05).

^*a*^Significant time effect.

^*b*^Significant time*group effect.

^*c*^Significant group effect.

## 4. Discussion

The advent of private space flights raises issues concerning the health of individuals who will compose the crews. While the future of space tourism is being built, short term space journeys pose a first challenge. These voyages will be characterized by repeated changes in gravity. Studying the impact of these repeated gravity changes on the psychophysiological and exteroceptive responses of these tourists is a key factor in ensuring the success of these missions. Knowledge of these impacts will enable countermeasures to be considered. Such knowledge can be acquired from parabolic flights, which provide an established model to measure effects of gravity changes. Therefore, our study has two main objectives: [1] to investigate the impact of space travel on psychophysiologcial and sensory responses using a parabolic flight experience; [2] to assess recovery from a parabolic flight experience one week later; [3] to evaluate whether a high parasympathetic functioning at baseline may constitutes a biomarker of adaptation for individuals unaware of any selection and spatial training.

Our study highlights major findings. First, the health of future space tourists does not appear to be at risk. Adaptation may be targeted using the parasympathetic activation of flyers at baseline. The HP profile had better psychophysiological and sensory responses (i.e., high parasympathetic functioning at baseline) postflight while they perceived more subjective stress and were more impacted on affect at recovery compared to the LP profile (i.e., low parasympathetic functioning at baseline). Second, flying experience ranging from first-time flyers (i.e., first flight), intermediate flyers (i.e., less than 10 flights) to expert flyers (i.e., more than 30 flights) had no impact on our results. This last result is important, as it highlights that regardless of the number of hours flown, the same changes in psychophysiological and sensory responses were generated. The quality of interoception does not depend on the subjects’ flying experience and may constitute an endophenotype characterizing the subjects’ response modality that could be accessible to simple countermeasures. Third, a significant decrease in the flyers’ psychophysiological state of functioning the day after the flight and up to one-week postflight underlines the need to implement strategies to mitigate these effects.

### 4.1. Implications of physiological profiles at baseline

At baseline, those with an HP profile have a better interoceptive sensitivity than those with an LP profile, which is further highlighted by a higher attitude of acceptance in the present moment, a lower ignorance or distraction from sensations of pain or discomfort, and a trend to more trust in their body sensations. This profile has been associated with emotional balance, well-being, fewer pathological disorders [76–81], and thus better health outcomes and self-awareness. Nevertheless, they perceived high subjective stress. This apparent contradictory response is not entirely irrelevant. It is important to note that parasympathetic activation does not necessarily mean a decreased ability to perceive stress. An active parasympathetic response may be a compensatory reaction to high levels of perceived stress, aimed at reducing the negative effects of stress on the body. The parasympathetic system is involved in stress regulation and participates in the individual’s adaptive response to external changes [40,41]. Thus, although the HP profile may show better physiological stress regulation, as well as a higher coping acceptance, they may still perceive subjective stress. In addition, there are complex interactions between the parasympathetic and sympathetic branches of the ANS [82]. The HP profile activates its response to a challenging situation and is in an acceptance situation of what they have experienced. Thus, this profile does not necessarily reflect complete inhibition of the sympathetic response. The HP profile may therefore have a finer modulation of autonomic regulation, which could enable them to perceive their subjective stress more clearly while maintaining a physiological stability. This result is linked to the physiological activation of the ANS.

Some HRV indicators highlighted a strong parasympathetic tone and effective autonomic regulation of the HP profile towards the LP profile (i.e., a higher SDNN, pNN50, LF, HF, SD1, SD2, SD ratio). This indicates that individuals with an HP profile have a better autonomic regulation, which can be beneficial in terms of stress resilience, recovery, and physiological balance [37,41,45]. Nevertheless, the HP profile shows a lower SD ratio and α2 index. The SD ratio is associated with the randomness of the HRV signal [83]. In the context of HRV, this might mean that the heart rate fluctuations are less structured or show reduced fractal-like behavior over time. This pattern could indicate that the HP profile has a regulatory strategy that prioritizes rapid adaptability and responsiveness, possibly due to their strong parasympathetic influence. Thus, the HP profile might be indicative of a finely tuned regulatory response that does not require as much broad-scale modulation compared to the LP profile. Both psychological and physiological responses highlight a specific regulatory pattern for the HP profile that contributes to their resilience and adaptability in the face of stressors. Altogether, these results show that stress acts bidirectionally on the brain-body axis [84,85]. Signals are encoded multimodally via interoceptive pathways, but also in an exteroceptive manner via peripheral receptors (i.e., visual, vestibular, somatosensory) [85,86,87]. The HP profile has a lower slope with eyes open and a lower NA02 with eyes closed than the LP profile. This result may reflect a better ability of the postural system to maintain stability and reduce excessive body oscillations in response to perturbations before a challenge. These results could be linked to better parasympathetic modulation, promoting finer control and greater stability during balance tasks on the stabilometric platform. The better interoceptive and proprioceptive abilities for the HP subjects suggest that they could have a better brain-body connection. Interoception may underline the relationship between cardiac and exteroceptive biosignal responses [88,89]. In a review, Pinna and collaborators [86] highlighted that higher HRV, especially the parasympathetic branch of the ANS, and higher interoception leads to increased emotional regulation. Especially the parasympathetic activity has been associated with better adaptative emotional regulation strategies. Thus, the regulatory loops involved in the interoceptive pathways participate in the homeostasis. Individuals with high-quality interoception and an autonomic response adjusted to environmental demand would be more attuned to the physiological changes that occur when exposed to a stressful situation. As a result, they would have greater adaptive capacities. These biomarkers could thus be relevant to the management of short- and long-term stressors, whatever their intensity, and enable better recovery. Craig et al. [77] show that vagal and spinal afferents may represent parasympathetic and sympathetic signal activities. These two branches of the ANS may inhibit each other into the interoceptive regions of the brain.

Thus, these results suggest a baseline endophenotype response between two profiles characterized by their level of parasympathetic functioning towards a better or less adaptative functioning.

### 4.2. Impact of repeated gravity changes on human factors

Our study sheds light on the impact of repeated gravity changes, including microgravity, on psychophysiological and exteroceptive responses. Psychological measures indicate that repeated gravity changes induce positive outcomes at postflight, except for postural stability. These interactions between the body and the environment play a fundamental role in how individuals act on their environment and cope with changes in performance [76,90]. The HP profile tends to have a better acceptance (i.e., non-reactivity to inner experience) of how things are in the present moment and without judgment than the LP profile. Moreover, they have a better interoceptive awareness. In more detail, they do not ignore or distract themself from sensations of pain or discomfort, experience their body as a safe and trustworthy place, tend to actively listen to the body for insight and tend to have a higher connection between body sensations and emotional states compared to the LP profile. Therefore, the HP profile would be more attentive and aware to the information received from the inner body and more able to live in the present moment. DeHart [91] highlighted that individuals must immediately accept the reality of the environment and react to their surroundings. Since the parabolic flight experience has no impact on psychophysiological and proprioceptive response, the future regular spaceflights to which space tourists will be subjected to will not be sufficient to induce long-term adaptation. While brain neuroplasticity enables the human body to efficiently adapt to changes in the environment in a few minutes, the quality of this adaptation long term will differ depending on the resources and reserves that the individual is able to mobilize, as well as their physical and mental preparation [91]. Subjective sleep ratings indicate that those with a HP profile have fewer physical indicators of a stress statebefore sleep than the LP profile, but this is also significant pre-flight. Moreover, flyers tend to perceive less subjective stress postflight. This statement is corroborated by the physiological measure that underline a higher HRV (i.e., RR intervals, SDNN, RMSSD, pNN50, HF) with a higher unpredictability and flexibility of the signal (i.e., SD1, SD2, SD ratio, α1) at postflight compared to baseline and pre-flight. Also, participants tend to increase their SampEn at postflight compared to baseline. Therefore, the flight leads to a decline both in psychological and physiological stress. The literature has linked these findings to a greater adaptability and better health [37,92,93]. Thus, flyers have an increase in the functioning of the sympathetic and parasympathetic components of the ANS, especially the HP profile.

Proprioception was deeply impacted during our experiment. Effects were observed in both closed and open eye conditions for slope, energy, logarithm in the left to right plan. More specifically, under open-eye conditions, the LP profile showed a trend towards a higher left/right logarithm, as well as a higher slope and a lower left/right velocity variance compared to the HP profile. This result may reflect a reduced symmetry or postural stability between the left and right sides of the body, and thus an altered postural balance. Moreover, at postflight, flyers showed an increase in slope (i.e., probably linked to tilt or displacement of the center of pressure) in both open-eye and closed-eye conditions compared to pre-flight, as well as a decrease in energy, but only in closed-eye conditions. This result may indicate a greater alteration in postural balance in the absence of visual references. The HP profile, meanwhile, appears to be the most affected, with a tendency for the slope to increase at postflight compared to pre-flight. Human beings are familiar with utilizing dependable visual, vestibular, and somatosensory cues to govern intricate balance processes. The linear acceleration of stimuli reaching the otolith receptors affects sensory inputs, playing a crucial role in spatial orientation on Earth. These signals are integrated with visual and vestibular inputs [94,95] and contribute to postural stability [96]. Therefore, the neurovestibular system is one of the first to be affected in space [97]. Homick and Reschke [98] have shown that postural stability has been decreased during parabolic flights. This effect was increased in the closed eyes condition. Sensorimotor performance took two weeks to return to their baseline score. In line with these findings, a study in the NEEMO habitat analog found that crew members had not recovered their pre-mission sensorimotor performance after several weeks [2]. Postural stability and control involve high-level cognitive functions, such as attention to bodily cues that provide information about the body’s orientation in space [99]. In this context, earlier work [100] demonstrates that the slope of the line that connects the center of pressure to the mean location index provides clinical information about potential pelvic torsion. At the same time, factors associated with postural instability have been shown to be associated with reduced sensation [101]. Furthermore, flyers have a better identification of odors postflight compared to baseline. Stress is known to increase the perception of odors. A study shows a link between cortisol and odor identification [102].

Overall, the parabolic flights induce a decrease in psychological and physiological stress but impaired postural balance. The HP profile may be protective, notably through a better interoceptive sensitivity and a higher parasympathetic functioning, compared to the LP profile, providing a more adaptative and flexible response behavior.

### 4.3. Need to prepare postflight experience by integrating the recovery to the space experience

The recovery period seems to be the one for which the impact of repeated changes in gravity, including microgravity, is major and persistent. Many individuals did not recover, or even deteriorated further. Sleep and emotions seem to be the most impaired components.

Flyers show an increase in coping flexibility from the day after and up to one-week postflight, reflected in the abandonment of strategies deemed ineffective for adaptation via a process of understanding one’s environment, monitoring and evaluating results, and abandoning the coping strategy when results are unfavorable. The lack of significance for the “adaptation” dimension reflects the absence of implementation of an alternative strategy. Coping flexibility makes it possible to respond to environmental demand in order to produce more adaptive outcomes [103]. Abandoning an ineffective strategy has been linked to protection against repeated failure, and thus orientation towards a depressive or potentially health-damaging state [104–106]. Similarly, Kato et al. [107] showed that the quit strategy was correlated with low psychological stress. Moreover, flyers’ tendency to ignore or distract themselves from sensations of pain or discomfort increases, and their ability to maintain and control their attention on their bodily sensations tends to decrease in postflight. They recovered one-week postflight, although the values had not yet returned to baseline.

In terms of affect, subjects experienced a drop in positive affect and an increase in negative affect the day after and up to one-week postflight compared to baseline and postflight. This result means that the experience of repeated changes in gravity induces very strong positive affect, with a return to reality that can be complex to cope with, following the extraordinary experience. A week after the end of the flights, the emotional impact is still present. The only difference in positive affect is linked to the parasympathetic functioning of the flyers. Regardless of profile, both had a decrease in positive affect. However, those with an HP profile showed the greatest decrease compared to those with an LP profile. Schneider et al. [49] demonstrated mood changes during a parabolic flight, reflecting an increase in environmental demand. Sleep monitoring revealed that flyers had higher wakefulness, reduced sleep efficiency and more nighttime awakenings the day after the flight. However, they felt they had a better night’s sleep than the pre-flight night on subjective ratings. Nevertheless, the HP profile had poorer sleep quality than the LP profile. These results are in line with the better interoceptive and cardiac biosignal functioning highlighted earlier. It’s worth noting that individuals report less repetitive thoughts and physical indicators of stress the day after the flight, and better sleep quality one-week postflight. Sleep disorders appear to have the most negative impact during missions [1]. Kanas [108] showed that a decrease in sleep quality is one of the main factors influencing neuropsychological changes. This result gives rise to two facts: the first relates to the intense preparation inherent in parabolic flights, which leads to greater fatigue, and the second to the need for recovery spread over a period of up to a week. Fried-Werner and collaborators [109] observed an association between sleep quality preflight and higher cortisol levels during parabolic flight. Moreover, our results showed that flyers tend to have a higher need for attention regulation to the body after the flight. This result may be related to the impact of parabolic flight on postural control and may suggest the need to recover countermeasures.

### 4.4. Perspectives for space tourism

In summary, our results demonstrate no major deterioration in psychophysiological functioning due to repeated changes in gravity, including microgravity. In all the flyers, the postflight phase undermines the interoceptive components, with a willingness to ignore internal information. Although not significant, we can observe that at one-week postflight the values have not returned to those measured at baseline. Even so, they are determined to regulate their attention to the body via postural awareness. Flyers seem to find a way to manage their psychic state and thus find a coping mechanism. Results show that those with high parasympathetic functioning are the ones for whom the after-experience is the most difficult to cope with, even though they have a higher level of interoceptive awareness than those with lower parasympathetic functioning. They also have poorer sleep quality. This HP profile reflects a greater awareness of the environment. They are aware of what’s going on around them, regardless of whether the information received is positive or negative. This seems to indicate a better adaptation. In terms of cardiac bio-signals, the results were in accordance with the challenge, with an increase in HRV at the end of the flight, but also via a coactivation of sympathetic and parasympathetic branches of the ANS, particularly in those with a HP profile compared with those with a LP profile.

Although our results do not suggest that repeated changes in gravity will constitute a major health risk, they underline that the experience of parabolic flight induces psychophysiological and sensory changes that may have consequences in everyday life. These changes are still present one-week postflight. These results reflect the need to take care of the after-experience of parabolic flights in the context of space tourism, especially for those with an HP profile. Thus, every training session should include preparation (i.e., coaching) for the aftermath, to avoid any psychological difficulties that might result, and to facilitate coping. The next flights will have to prepare private crews by following a rigorous training program. The latter will have to follow the latest advances in personalized medicine, in order to offer a program tailored as closely as possible to the needs of each individual. This will be the challenge of the next few years. It has become urgent to establish a framework to regulate space tourism from the point of view of health behaviors. Postflight training programs will need to include modules designed primarily to strengthen cognitive and emotional resources, and even to introduce systematic monitoring of crews during the postflight weeks. Pre-flight medical screening will be pre-determined and requires the utmost attention. A blank canvas can be used to build comprehensive modules for physical preparation, without forgetting the psychological and cognitive dimensions. These courses may include a theoretical framework, practical modules for learning soft skills essential to crew safety (e.g., communication, cohesion, teamwork) and techniques for managing emotional and cognitive stress levels (e.g., cardiac coherence, meditation, games), training to increase the body’s resistance in centrifuges, hypobaric chambers, parabolic flight, drop towers and any other environment that may be analogous to orbital missions. Finally, a postflight preparation module will be essential to limit any psychophysiological disorders that may result. There is a need to target countermeasures tailored to individual profiles, paving the way for personalized preparation for space travel.

Overall, these results contribute to our understanding of the real human challenge of adapting to the constraints imposed by space travel. Who among us could have imagined 100 years ago that the most common of mortals could find himself in orbit? Looking up at the stars and wishing to reach them will no longer be a dream, but a possibility. We are pushing aside the boundaries of reality, turning the most universal of dreams into reality. Further research is therefore warranted to better understand typical stress environments, to highlight their impact on the human body and behavior, and to understand how humans adapt to them against all odds. It is essential not only to better manage the risks of disorders prior to departure on deep space missions, but also to propose relevant and effective countermeasures to significantly reduce them.

## 5. Limitations

This study has several methodological shortcomings that are inherent to the ecological environment. First, the paradigm in which we operate is space tourism, in which individuals will undergo repeated changes of gravity. These changes will not be as rhythmic as in a parabolic flight campaign, with a limited flight time of approximately 3 hours. The reality of the flight plan will therefore be different. Nevertheless, there is no paradigm closer to what space experiences will be like for tourists today. Second, the small sample size, and an imbalance between male and female, and right- and left-handed subjects are other limitations of the study. Studying such a population is complex, both in terms of time constraints, and access to infrastructure and personnel (operational constraints, attendance). Both the scientific team and participants must be flexible to run such an experiment. Third, our results are not reproducible beyond the specific experimental conditions and cannot be generalized. Fourth, psychological and interoceptive data (collected through questionnaires) are subjective measures. Intelligent sensors would provide more objective measures of subjects’ adaptation to extreme environments. Moreover, while the PolarH10 belt is currently the best tool to accurately record RR intervals with the minimum of artifacts given our environmental constraints, the device cannot replace an ECG.

## 6. Conclusion

The present study is one of the first to describe the impact of space travel induced by a parabolic flight experience on psychophysiological and sensorial responses. These results raise the question of the risks that may be induced by space tourism. They highlight three major findings: [1] the health of future space tourists does not appear to be at risk, as long as the individuals involved are healthy (e.g., with no pathologies that would make them unfit for flight), and adaptation may be improved regarding the parasympathetic activation; [2] the level of experience had no impact on the psychophysiological and sensory responses; and [3] the need to take care of crews over several days or even weeks and/or include a module to prepare them for the postflight period and the return to life on Earth. This study is the first to explore the psychological responses to one-week recovery from parabolic flight. The importance of preparation not only allows us to reflect on the preparation of tomorrow’s regular trips, with a population likely to be relatively heterogeneous, but also to target countermeasures adapted to individual profiles, opening the way to personalized preparation for space travel. Beyond this, these results contribute to our knowledge of the real human challenge of confronting the constraints of space travel.

## Supporting information

S1 Text Supplementary material. (DOCX)

## Abbreviations

α1: detrended fluctuation analysis self-similarity parameter that represented short-term fluctuations
α2: detrended fluctuation analysis self-similarity parameter that represented long-term fluctuations
ANS: autonomic nervous system
CFS: coping flexibility scale
ETOC: European Test of Olfactory Capabilities
FMI: freiburg mindfulness inventory
HP: high parasympathetic
HRV: heart rate variability
HF: high-frequency
ISS: international space station
LF: low-frequency
LFS: length ratio of the surface
LP: low parasympathetic
MAIA: multidimensional assessment of interoceptive awareness
NA02: maximum distance covered by the center of pressure
PAS: postural awareness scale
PSS: perceived stress scale
pNN50: percentage of adjacent NN intervals that differ from each other by more than 50 ms
RMSSD: root mean square of successive differences
RR: successive variation of the intervals between two heartbeats
SampEn: sample entropy achieving the regularity and complexity of time series
SD1: standard deviation of instantaneous short-term RR variability
SD2: standard deviation of continuous long-term RR variability
SDNN: standard deviation of the normal-to-normal RR interval
SPANE: scale of positive and negative experience
SQS: sleep quality scale
WASO: wakefulness after sleep onset
