# Supplementary material for: Effects of repeated gravity changes during parabolic flight: Evidence of the need to assist space tourists to outer space
Source: PLoS One. 2025 Apr 23;20(4):e0320588. doi: 10.1371/journal.pone.0320588 (PMC12017518; doi:10.1371/journal.pone.0320588)
Supplement: S1 Text — (DOCX) [file pone.0320588.s001.docx]

**Supplementary Material**

*Coping*. None of the adaptation dimension for time (*p* = .348, BF_10_ = .438), time*group (*p* = .168, BF_10_ = .320) and group (*p* = .832, BF_10_ = .708) reach significance. For evaluation dimension, time*group (*p* = .168, BF_10_ = .635) and group (*p* = .851, BF_10_ = .574) did not change.

*Mindfulness disposition*. No significant change were found for presence dimension at time (*p* = .381, BF_10_ = .324), time*group (*p* = .885, BF_10_ = .163), group (*p* = .840, BF_10_ = .488); and for acceptance dimension at time (*p* = .965, BF_10_ = .101), time*group (*p* = .523, BF_10_ = .147).

*Interoception*. None of the differences in the following interoceptive measures reached significance: noticing at time (*p* = .668, BF_10_ = .145), time*group (*p* = .381, BF_10_ = .128), group (*p* = .194, BF_10_ = .956); not distracting at time*group (*p* = .129, BF_10_ = 1.000); not worrying at time (*p* = .637, BF_10_ = .130), time*group (*p* = .274, BF_10_ = .073), group (*p* = .461, BF_10_ = .558); attention regulation at time*group (*p* = .186, BF_10_ = .855), group (*p* = .213, BF_10_ = .934); emotional awareness at time (*p* = .528, BF_10_ = .425), time*group (*p* = .721, BF_10_ = .244); self-regulation at time (*p* = .579, BF_10_ = .115), time*group (*p* = .767, BF_10_ = .149), group (*p* = .088, BF_10_ = 1.000) ; body listening at time (*p* = .230, BF_10_ = .242), time*group (*p* = .951, BF_10_ = .349), and trusting at time (*p* = .257, BF_10_ = .188), time*group (*p* = .797, BF_10_ = .313).

*Motion sickness*. Neither of the following measures at each timepoint: nauseas for time (*p* = .464, BF_10_ = .460), time*group (*p* = .464, BF_10_ = .196), group (*p* = .908, BF_10_ = .460); dizziness of eyes for time (*p* = .610, BF_10_ = .382), time*group (*p* = .610, BF_10_ = .167), group (*p* = .485, BF_10_ = .456); vertigo for time (*p* = .694, BF_10_ = .359), time*group (*p* = .694, BF_10_ = .150), group (*p* = .728, BF_10_ = .398) changed significantly.

*Emotion*. Positive affect did not change significantly for group (*p* = .306, BF_10_ < .000), and negative affect for time*group (*p* = .907, BF_10_ = .865) and group (*p* = .166, BF_10_ < .000).

*Sleep*. None of the following subjective sleep measures reach significance: sleep quality at time*group (*p* = .911, BF_10_ = 1.000); well rested waking up morning at time*group (*p* = .270, BF_10_ = .543) and group (*p* = .410, BF_10_ = .266); good quality of sleep at time (*p* = .096, BF_10_ = 1.000), time*group (*p* = .405, BF_10_ = .590) and group (*p* = .490, BF_10_ = .432); vivid dreams at time (*p* = .613, BF_10_ = .344), time*group (*p* = .708, BF_10_ = .416) and group (*p* = .136, BF_10_ = 1.000); frequently woke up at time (*p* = .131, BF_10_ = 1.000), time*group (*p* = .597, BF_10_ = .699) and group (*p* = .185, BF_10_ = 489); repetitive thoughts before bed at time*group (*p* = .238, BF_10_ = .741) and group (*p* = .384, BF_10_ = 143).

None of the following physiologic sleep measures reach significance: WASO at time*group (*p* = .937, BF_10_ = .875) and group (*p* = .227, BF_10_ = 221); sleep efficiency at time*group (*p* = .793, BF_10_ = .885) and group (*p* = .217, BF_10_ = 293); wake period during night at time (*p* = .084, BF_10_ = 1.000), time*group (*p* = .834, BF_10_ = .759) and group (*p* = .217, BF_10_ = 499); actual sleep time at time*group (*p* = .245, BF_10_ = .693) and group (*p* = .432, BF_10_ = .089); actual wake time at time*group (*p* = .523, BF_10_ = .778) and group (*p* = .345, BF_10_ = .192).

*Subjective stress*. Perception of stress did not change significantly at each time point: time (*p* = .991, BF_10_ = .083), time*group (*p* = .757, BF_10_ = .081) and group (*p* = .148, BF_10_ = .981).

*Postural awareness*. None of the ease/familiarity with postural awareness at time (*p* = .317, BF_10_ = .514), time*group (*p* = .688, BF_10_ = .275), group (*p* = .185, BF_10_ = .833) or need for attention regulation with postural awareness at time*group (*p* = .615, BF_10_ = .798), group (*p* = .259, BF_10_ = .367) indices changed significantly.

*Olfaction*. No significant change was found for detection at time (*p* = .717, BF_10_ = .341), time*group (*p* = .415, BF_10_ = .224), group (*p* = .712, BF_10_ = .620); identification at time*group (*p* = .166, BF_10_ = .607), group (*p* = .922, BF_10_ = .323), and hedonic value at time (*p* = .450, BF_10_ = .424), time*group (*p* = .710, BF_10_ = .343), group (*p* = .366, BF_10_ = .775).

*Proprioception*. In the opened eyes condition, none of the following differences were significant: logarithm at time (*p* = .785, BF_10_ = .344), time*group (*p* = .182, BF_10_ = .147) and group (*p* = .594, BF_10_ = .436); ellipse at time (*p* = .117, BF_10_ = 1.000), time*group (*p* = .529, BF_10_ = .485) and group (*p* = .559, BF_10_ = .403); slope at time (*p* = .180, BF_10_ = .306), and group (*p* = .259, BF_10_ = .187); LFS at time (*p* = .152, BF_10_ = .994), time*group (*p* = .313, BF_10_ = .433) and group (*p* = .658, BF_10_ = .429); average speed at time (*p* = .910, BF_10_ = .323), time*group (*p* = .177, BF_10_ = .161) and group (*p* = .467, BF_10_ = .473); speed variance at time (*p* = .716, BF_10_ = .333), time*group (*p* = .124, BF_10_ = .162) and group (*p* = .466, BF_10_ = .468); logarithm left to right at time (*p* = .976, BF_10_ = .324), and time*group (*p* = .228, BF_10_ = .339); range left to right at time (*p* = .449, BF_10_ = .421), time*group (*p* = .135, BF_10_ = .224) and group (*p* = .316, BF_10_ = .557); variance left to right at time (*p* = .086, BF_10_ = 1.000), time*group (*p* = .107, BF_10_ = .709) and group (*p* = .200, BF_10_ = .592); speed variance left to right at time (*p* = .618, BF_10_ = .329), and time*group (*p* = .205, BF_10_ = .335); NA02 left to right at time (*p* = .809, BF_10_ = .397), time*group (*p* = .583, BF_10_ = .228) and group (*p* = .206, BF_10_ = .696); logarithm front to rear at time (*p* = .742, BF_10_ = .365), time*group (*p* = .179, BF_10_ = .156) and group (*p* = .790, BF_10_ = .406); range front to rear at time (*p* = .274, BF_10_ = .675), time*group (*p* = .523, BF_10_ = .317) and group (*p* = .522, BF_10_ = .458); variance front to rear at time (*p* = .353, BF_10_ = .516), time*group (*p* = .383, BF_10_ = .261) and group (*p* = .426, BF_10_ = .511); speed variance front to rear at time (*p* = .958, BF_10_ = .343), time*group (*p* = .153, BF_10_ = .135) and group (*p* = .964, BF_10_ = .408); NA02 front to rear at time (*p* = .854, BF_10_ = .331), time*group (*p* = .571, BF_10_ = .249) and group (*p* = .188, BF_10_ = .751).

In the closed eyes condition, none of the following differences were significant: logarithm at time (*p* = .218, BF_10_ = .721), time*group (*p* = .790, BF_10_ = .474) and group (*p* = .222, BF_10_ = .640); ellipse at time (*p* = .146, BF_10_ = .897), time*group (*p* = .837, BF_10_ = .700) and group (*p* = .263, BF_10_ = .838); slope at time*group (*p* = .601, BF_10_ = .461), and group (*p* = .856, BF_10_ = .005); LFS at time*group (*p* = .145, BF_10_ = .591) and group (*p* = .448, BF_10_ = .264); average speed at time (*p* = .132, BF_10_ = 1.000), time*group (*p* = .880, BF_10_ = .444) and group (*p* = .673, BF_10_ = .438); speed variance at time (*p* = .374, BF_10_ = .484), time*group (*p* = .612, BF_10_ = .241) and group (*p* = .382, BF_10_ = .492); logarithm left to right at time (*p* = .603, BF_10_ = .177), and time*group (*p* = .867, BF_10_ = .397); range left to right at time (*p* = .108, BF_10_ = 1.000), time*group (*p* = .936, BF_10_ = .882) and group (*p* = .184, BF_10_ = .934); variance left to right at time (*p* = .169, BF_10_ = .727), time*group (*p* = .947, BF_10_ = .616) and group (*p* = .240, BF_10_ = .796); speed variance left to right at time (*p* = .601, BF_10_ = .327), time*group (*p* = .852, BF_10_ = .370), and group (*p* = .083, BF_10_ = 1.000); NA02 left to right at time (*p* = .396, BF_10_ = .495), time*group (*p* = .650, BF_10_ = .266) and group (*p* = .739, BF_10_ = .451); logarithm front to rear at time (*p* = .149, BF_10_ = 1.000), time*group (*p* = .761, BF_10_ = .465), and group (*p* = .563, BF_10_ = .434); range front to rear at time (*p* = .381, BF_10_ = .461), time*group (*p* = .862, BF_10_ = .215) and group (*p* = .971, BF_10_ = .494); variance front to rear at time (*p* = .517, BF_10_ = .386), time*group (*p* = .960, BF_10_ = .213) and group (*p* = .786, BF_10_ = .527); speed variance front to rear at time (*p* = .189, BF_10_ = .862), time*group (*p* = .974, BF_10_ = .356) and group (*p* = .728, BF_10_ = .420); NA02 front to rear at time (*p* = .214, BF_10_ = .608), time*group (*p* = .080, BF_10_ = .426) and group (*p* = .130, BF_10_ = .669).

*HRV*. RR intervals at time*group (*p* = .347, BF_10_ = .714), group (*p* = .355, BF_10_ = .001); SDNN at time*group (*p* = .863, BF_10_ = 1.000); SDNN at time*group (*p* = .863, BF_10_ = 1.000); RMSSD at time*group (*p* = .378, BF_10_ = 1.000); LF at time (*p* = .262, BF_10_ = .130), time*group (*p* = .897, BF_10_ = .339); LF/HF ratio at time (*p* = .527, BF_10_ = .633), time*group (*p* = .068, BF_10_ = .122), group (*p* = .801, BF_10_ = .478); SD1 at time*group (*p* = .378, BF_10_ = 1.000); SD2 at time*group (*p* = .880, BF_10_ = 1.000); SD ratio at time*group (*p* = .431, BF_10_ = 1.000); α1 at time*group (*p* = .687, BF_10_ = .755), group (*p* = .213, BF_10_ = .066); α2 at time (*p* = .664, BF_10_ = .039); and sample entropy at time*group (*p* = .157, BF_10_ = .412), group (*p* = .959, BF_10_ = .304) did not change significantly.
